# Bias in Structural MRI Correlates of Delay Discounting due to Head Motion

**DOI:** 10.1002/hbm.70474

**Published:** 2026-02-22

**Authors:** James Read‐Tannock, Andrew Reid, Etienne Farcot, Martin Schürmann, Christopher R. Madan

**Affiliations:** ^1^ School of Psychology University of Nottingham Nottingham United Kingdom; ^2^ School of Social and Behavioural Sciences Tilburg University Tilburg the Netherlands; ^3^ School of Mathematical Sciences University of Nottingham Nottingham United Kingdom

## Abstract

A growing number of studies report that delay discounting, a measure used to evaluate impulsive behaviour, is associated with the regional volume or thickness of cortical grey matter. Using 1096 participants' MRI data from the open Human Connectome Project Young Adults dataset, we show that delay discounting correlates significantly with in‐scanner head motion, a prevalent cause of artifacts in magnetic resonance imaging. Furthermore, head motion was found to bias estimates of grey matter thickness downwards across much of the neocortex, including many of the regions where delay discounting has been associated with reduced grey matter, suggesting these effects may be confounded by motion artifacts. The effects associated with delay discounting were also significantly correlated with the cortical thinning effects associated with motion under a spin permutation null model. In conclusion, we suggest that any future studies investigating structural correlates of delay discounting should correct for motion effects—either during data acquisition or by including a movement covariate when fitting models.

## Graphical abstract

Associations of delay discounting and head motion with cortical thickness. (A) Summary of previous literature findings as having altered cortical thickness with delay discounting behaviour. (B) Results for reductions in cortical thickness correlating with delay discounting and head motion, using parcellated regions. (C) Results for reductions in cortical thickness, analysed vertex‐wise analyses.

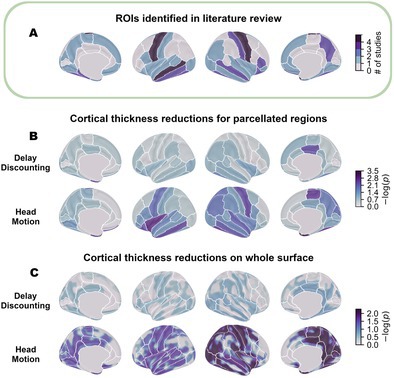

## 1 Introduction

Delay discounting is a commonly employed measure of future‐oriented or impulsive behaviour that has been linked to altered cortical structure in a number of studies. In this task, participants are asked questions such as ‘Would you prefer £200 today or £250 in one month?’. The resulting measure exhibits high test–retest reliability and pertains to how much an individual discounts the value of future rewards (Kirby and Maraković 1996; Odum 2011). In other words, it is a measure of time preference, that is, how much immediate gratification is preferred to delayed gratification. Given the sometimes extended duration of MRI scans and links of other measures of impulsivity to increased in‐scanner motion (Kong et al. 2014), we investigated whether delay discounting may also correlate with increased movement and if this may account for the differences in cortical thickness measured in structural MRI.

Motion artifacts are a significant challenge in structural magnetic resonance imaging (MRI) of the brain, often compromising the quality of the images obtained. Participant head motion has been shown to bias quantitative estimates of brain morphology derived from structural MRI, even after manual quality control procedures (Alexander‐Bloch et al. 2016; Elyounssi et al. 2025; Madan 2018; Pardoe et al. 2016; Reuter et al. 2015; Savalia et al. 2017), and methods used to control for head motion in functional MRI are not directly applicable to structural scans. However, studies of relationships between brain structure and behaviour often overlook the confounding effect of head motion on estimates of brain structure. Head motion is highly correlated across scanning sessions and is a stable and heritable behavioural trait which has previously been shown to correlate with body mass index (BMI), age, delay discounting and factors such as history of smoking (Beyer et al. 2017; Couvy‐Duchesne et al. 2014; Engelhardt et al. 2017; Hodgson et al. 2017; Madan 2018; Zeng et al. 2014). This is problematic when specific populations of interest exhibit increased head movement, for example in clinical conditions such as schizophrenia and Parkinsonism (Makowski et al. 2019; Pardoe et al. 2016). This confound may be a contributing factor to the poor reproducibility of brain‐behaviour relationships reported previously (Boekel et al. 2015; Kharabian Masouleh et al. 2019, 2022; Marek et al. 2022). Ideally, prospective motion correction (Dosenbach et al. 2017; Frost et al. 2019; Lüsebrink et al. 2017; Maclaren et al. 2013; Tisdall et al. 2012; White et al. 2010), which detects and corrects for head movement during structural scans, should generally be integrated into structural MRI sequences where quantitative analysis of cortical morphology is anticipated in order to improve data quality and minimise spurious effects on brain morphology metrics (as well as to reduce costs of re‐acquisition when images are unusable; see Slipsager et al. 2022).

Here, we used data from the Human Connectome Project‐Young Adults 1200 Subjects dataset (HCP‐YA S1200; Van Essen et al. 2013), a large open‐access neuroimaging project that includes structural and functional MRI, for which prospective motion correction was not used (HCP S1200 Reference Manual 2018, 34; Van Essen et al. 2013). Many studies have used the extensive battery of behavioural tests collected in the HCP in conjunction with the comprehensive imaging dataset to study brain‐behavioural associations (reviewed in Madan 2022), but generally without taking account of movement‐related confounds. Using estimates of head movement/in‐scanner motion derived from the functional MRI data of the same session, as used and validated in previous studies (Alexander‐Bloch et al. 2016; Engelhardt et al. 2017; Savalia et al. 2017), we investigate the effects of head motion on estimates of cortical morphometry in the HCP‐YA S1200.

A recent study by Kharabian Masouleh et al. (2022) using the same dataset showed that brain‐behaviour associations discovered in small samples generally failed to replicate in larger ones. Notably, they found that delay discounting—a behavioural metric often related to impulsivity—had the second most replicable relationship with cortical thickness in held‐out test data, only exceeded by the effects of age. Given age‐related cortical thinning is such a well‐established result (Fjell et al. 2009; Madan and Kensinger 2016, 2018; Salat et al. 2004), this warrants some attention and raises the question of why delay discounting, in particular, is associated with widespread and robust variability in cortical thickness above other psychological measures examined (e.g., working memory (image‐based 2‐back) and the five‐factor model of personality traits).

**FIGURE 1:**
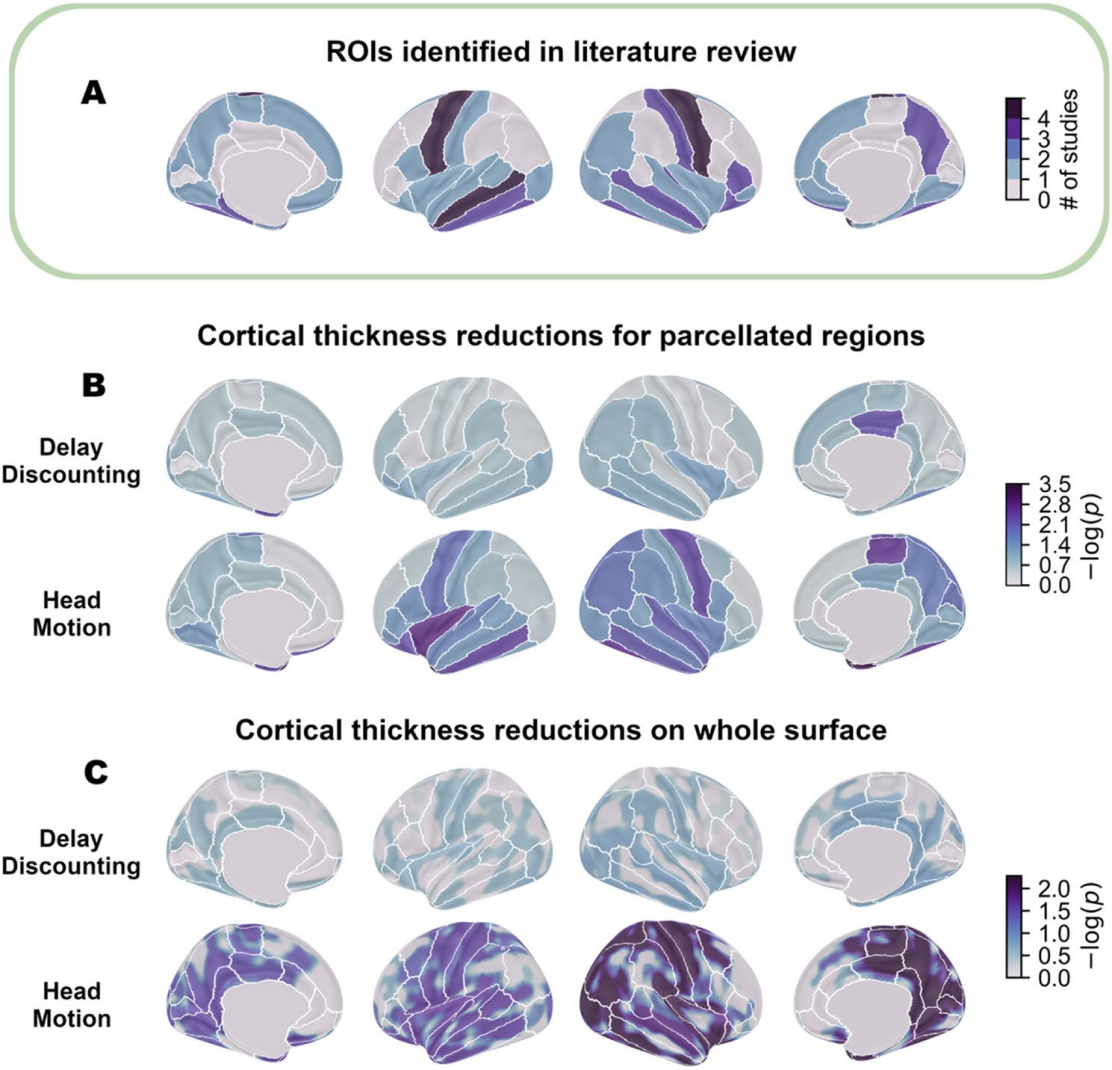
Associations of delay discounting and head motion with cortical thickness. (A) Summary of previous literature findings, plotted using the Desikan‐Killiany parcellation identified in 5 studies with comparable methods from the literature (see Table 1) as having altered cortical thickness with delay discounting behaviour. (B) Results from region‐wise permutation testing for reductions in cortical thickness correlating with delay discounting mAUC and head motion at each parcellated ROI. Values underlying this figure are in Table S1. (C) Results from vertex‐wise analysis of cortical thickness with 15 mm FWHM smoothing kernel applied. Colour intensity represents *p*‐values for the variable of interest, base‐10 log‐transformed for visualisation purposes.

Numerous studies have reported putative links between cortical morphology and delay discounting, often finding reduced thickness and/or volume in a broad array of regions, a selection of which are summarised in Table 1 (also see Figure 1A). Most use automated pipelines for estimating brain morphology, such as FreeSurfer (Barry et al. 2020; Bounoua et al. 2023; Garzón et al. 2023; Grodin et al. 2017; Ho et al. 2016; Owens et al. 2017; Pehlivanova et al. 2018; Sadeh et al. 2023; Westwater et al. 2019), which uses an iterative process to reconstruct a 3D cortical surface and automatic methods for aligning the obtained surfaces to a normative template (Dale et al. 1999; Fischl, Sereno, and Dale 1999; Fischl, Sereno, Tootell, and Dale 1999). Estimates of the thickness, surface area and grey‐matter volume (GMV) are then calculated from the reconstructed white matter and pial (grey) matter surfaces.

**TABLE 1:** Delay discounting morphometry studies.

Study	Method	*N*	Morphometric variable	Dependent variable	Associated regions
Sadeh et al. (2023) *Psychological Medicine*	Fitted value of delay discounting based on 148 FreeSurfer parcellated ROIs	1105 (HCP Young Adults) + 152 + 73	Cortical thickness	ln(k)	N/A. No significance tests reported
Bounoua et al. (2023) *PLoS One*	Validating weights from Sadeh et al. (2023) on adolescent dataset with regression analysis.	9992 + 56	Cortical thickness	ln(k)	N/A
Westwater et al. (2019) Developmental Cognitive Neuroscience*	Regression on inferior frontal gyrus regions cortical thickness from FreeSurfer	75 + 75	Cortical thickness	AUC	Inferior frontal gyrus (pars opercularis)
Lempert et al. (2020) *Neuropsychologia*	Regression on medial temporal lobe regions cortical thickness from ‘ASHS‐T1’ automatic segmentation pipeline.	100	Cortical thickness	ln(k)	Entorhinal
Pehlivanova et al. (2018) Journal of Neuroscience*	Generalised additive model regression on Advanced Normalisation Tools (ANTs). Including extensive image quality control	427	Cortical thickness	ln(k)	Frontal pole, medial orbital, central operculum, temporal pole, superior temporal, precentral, left medial frontal, left fusiform, right middle temporal, right inferior occipital, right cuneus, right posterior insula
Garzón et al. (2023) Cerebral Cortex*	Linked ICA across metrics from FreeSurfer and FSL‐VBM	1038 (HCP Young Adults)	Cortical thickness, surface area, and GMV	ln(k)	Top ICA loadings: temporal pole, precuneus, lingual, postcentral, temporal, parahippocampal, fusiform, precentral
Wang et al. (2016) *NeuroImage*	Multivariate pattern analysis on GMV (FSL‐VBM)	227	GMV	ln(k)	Right frontal pole, left middle frontal, left orbitofrontal, right parahippocampal, right precentral, left temporal pole, precuneus
Matsuo et al. (2009) *Human Brain Mapping*	VBM using SPM FDR correction	62	GMV	Barratt Impulsiveness Scale	Right middle orbitofrontal, left superior orbitofrontal, left anterior cingulate
Owens et al. (2017) NeuroImage*	Partial correlations with parcellated GMV derived from FreeSurfer surface reconstruction	1038 (HCP Young Adults)	GMV	AUC	Entorhinal, middle temporal, inferior temporal, precentral, lateral orbitofrontal, left lateral occipital, left postcentral, left precuneus, left insula, left transverse temporal, left temporal pole, right fusiform, right banks of the superior temporal sulcus, right superior frontal, right parahippocampal
Barry et al. (2020) Schizophrenia Research*	FreeSurfer vertex‐wise mri_glmfit with—sim option	174	Cortical thickness	ln(k)	Left temporal gyrus, left superior frontal, left pars triangularis, left pars orbitalis, precentral, paracentral, caudal middle frontal
Bjork et al. (2009) *Biological Psychiatry*	Manual segmentation, proportional volume used in linear models controlling for age	29	GMV	ln(k)	Inferolateral frontal, dorsolateral frontal
Grodin et al. (2017) *Drug and Alcohol Dependence*	FreeSurfer parcellated grey matter (ICV normalised) volume and thickness	109	GMV and cortical thickness	ln(k) and Barratt Impulsiveness Scale	Anterior insula (volume and thickness). Anterior cingulate cortex (volume only)
Guo et al. (2017) *Neuropsychologia*	VBM in SPM8	36	GMV	AUC	Clusters in ventral posterior cingulate and ventromedial prefrontal
Ho et al. (2016) *Behavioural Brain Research*	FreeSurfer parcellated white matter volume. MANCOVA	89	White matter volume	AUC	Orbitofrontal, dorsolateral prefrontal, medial prefrontal, motor

*Note:* Review of literature linking cortical morphometry to delay discounting or other measures of impulsiveness. The regions from studies marked with an asterisk (*) are included in Figure 1A. Where studies used more than one sample this is denoted with a + sign in the *N* column. Areas are bilateral unless stated otherwise. AUC and ln(k) are two metrics of delay discounting behaviour determined from the individual's indifference function.

Abbreviations: AUC: area under the curve, valued between 1 and 0 where lower values imply greater delay discounting; ln(k): log‐transformed hyperbolic discounting k parameter, generally negative‐valued with higher values implying greater delay discounting. GMV: grey‐matter volume.

If individuals with high delay discounting rates display increased in‐scanner head motion, this would contribute to reduced estimates of cortical thickness from in vivo structural MRI. Previous work has shown correlations between head movement and delay discounting (Hodgson et al. 2017) or other measures of impulsivity (Kong et al. 2014), but this is not considered in many recent studies examining brain structure and delay discounting. To assess the existing literature on brain structure and delay discounting, we conducted a thorough search and found 14 studies (see Table 1). Notably, none of the studies we reviewed had any correction of structural measures for head movement, and though quality control (QC) methods were commonly applied, this only results in excluding grossly affected scans, not correcting the more subtle artifacts affecting quantitative morphology estimates (Reuter et al. 2015). Therefore, we aimed to reproduce these previous findings using the large HCP S1200 dataset. However, unlike some earlier studies on the same dataset, we use methods suited to the family structure (twins and siblings) present in this sample. Because correlations have mostly been shown on FreeSurfer processed data both in terms of parcellated ROIs and at the vertex level using cluster detection algorithms, we decided to use both of these approaches here.

In this study, we therefore chose to investigate the following research questions: first, could we replicate previous findings connecting delay discounting with reductions in cortical thickness and/or grey‐matter volume (GMV)? Second, could we replicate previous findings connecting in‐scanner motion with reductions in cortical thickness and/or GMV? Finally, and most importantly, to assess the risk of a confound: how similar is the spatial distribution of effects from delay discounting and head motion?

## 2 Methods

### 2.1 Structural MRI Data Acquisition

Structural MRI images were acquired by the HCP team using a custom 3 T Siemens Skyra scanner located at Washington University in St Louis (Van Essen et al. 2013). T1‐weighted images were acquired using a 3D MPRAGE sequence with 0.7 mm isotropic resolution (TR = 2400 ms, TE = 2.14 ms, TI = 1000 ms, Echo Spacing (ES) = 7.6 ms) and T2‐weighted images using a variable flip angle turbo spin‐echo sequence (Siemens SPACE) at the same resolution (TR = 3200 ms, TE = 565 ms). Both T1w and T2w images were acquired using the parallel imaging technique GRAPPA implemented in the Siemens software with an acceleration factor of 2. The T1‐ and T2‐weighted images were then corrected for gradient nonlinearity distortions specific to the custom scanner before further processing, see Glasser et al. (2013) for further details.

An optical motion tracking system (Moire Phase Tracker, Kineticor) was mounted in the scanner but that data is not shared in the HCP dataset. Head motion from this system would have been a preferable measure of head motion, rather than estimation from the fMRI time‐series (see Section 2.4). Moreover, prospective motion correction methods were not yet validated at the time that data collection began (Van Essen et al. 2013).

For quality control, each structural scan was visually evaluated and rated for image quality (poor, fair, good, excellent). Scans with a poor or fair rating were then re‐acquired (Van Essen et al. 2013). Where more than one high‐quality T1w or T2w scan was obtained (indicated by the T1_Count and T2_Count variables in the HCP dataset), they were registered to each other using a rigid‐body transform and averaged before further processing (Glasser et al. 2013).

### 2.2 FreeSurfer Cortical Morphometry

Both the T1w and T2w images were processed by the HCP using a modified FreeSurfer version 5.2 *recon‐all* pipeline, which generated the cortical thickness and GMV estimates used in this study (please refer to Glasser et al. 2013 for more details).

For this analysis, we used only data from the 1096 participants who had task‐based functional MRI data which could be used for the multi‐modal surface‐matching registration algorithm MSM‐All (Glasser et al. 2016) and for estimating in‐scanner head movement during the structural MRI scan (see section on ‘Head Motion’ below). We downloaded and used the pre‐processed structural MRI data for these participants, but we estimated intracranial volume (ICV) separately from the native space T1 volumes using SPM12 segmentation (Lorio et al. 2016) because the FreeSurfer estimates of ICV contained outliers and increased variability towards the lower end (see Figure S1). Previous work has found FreeSurfer estimates of ICV to be biassed (Klasson et al. 2018; Nordenskjöld et al. 2013), while the new segmentation algorithm introduced since SPM8 has been shown to closely correspond with manual ICV measurements (Malone et al. 2015). More generally, FreeSurfer test‐retest reliability for cortical morphometry is quite high (see Madan and Kensinger 2017).

### 2.3 Delay Discounting Behaviour

The delay discounting task used in the HCP is an adjusting‐amount approach (based on Estle et al. 2006) designed to quickly find indifference points (i.e., the amount of immediate reward that is deemed equivalent to the larger, delayed reward). For each specified time period (1 month, 6 months, 1 year, 3 years, 5 years and 10 years) the participant is asked whether they would prefer half the reward immediately, or the full reward at the end of the period. Depending on the initial answer, the immediate reward is adjusted up or down and this continues for 5 iterations, with the final value being taken as the indifference point. This procedure was performed for both $200 and $40,000 as the full reward amount. The measure of delay discounting given in the HCP data is area under the curve (AUC; Myerson et al. 2001), where an AUC value of 1 indicates that the individual values rewards completely independent of when they will receive them (i.e., there is no delay discounting). Therefore, lower AUC values are conventionally associated with greater impulsivity. In this study, following Owens et al. (2017), we average the results for both reward amounts to produce mean area under the curve (mAUC). More details about the experiment are available in the HCP S1200 Data Release Manual (WU‐Minn Consortium of the NIH Human Connectome Project 2018).

Participants were excluded if delay discounting results were inconsistent, following Owens et al. (2017). Specifically, if the indifference point for a given time period was lower than for one of the preceding, shorter time periods, it was counted as inconsistent, and participants were excluded if there were 3 or more inconsistencies. Fifty‐nine participants were excluded on this basis before any further analysis was performed, bringing the total sample size to 1037 individual participants.

### 2.4 Movement Estimation

In the absence of head motion measurements from the structural scans, we used fMRI head movement time courses as a proxy for structural MRI head movement, following earlier work (Alexander‐Bloch et al. 2016; Savalia et al. 2017). The parameters resulting from a rigid‐body transformation of each fMRI volume to the reference image are included for each fMRI recording in the HCP S1200 dataset, and consist of translations and rotations in the x, y and z directions. We calculated framewise displacement according to the formulas used in Power et al. (2012):

$$\Delta d_{\mathrm{ix}}=d_{\left(i-1\right)x}-d_{\mathrm{ix}}$$

$$FD_{i}=\left|\Delta d_{\mathrm{ix}}\right|+\left|\Delta d_{\mathrm{iy}}\right|+\left|\Delta d_{\mathrm{iz}}\right|+\left|\Delta \alpha_{i}\right|+\left|\Delta \beta_{i}\right|+\left|\Delta \gamma_{i}\right|$$

where *d*_*ix*_ signifies the translation parameter for the *x* direction in frame *i* of the timeseries, and the three rotation parameters *α, β* and *γ* are first converted from degrees to millimetres by calculating the arc length on a circle with radius 50 mm (to approximate the size of the brain):

$$\mathrm{arc}\;\text{length}=\frac{\theta }{180}\mathrm{\pi r},$$

where θ is the angle in degrees and *r* is the radius in millimetres.

Framewise displacement was then divided by the repetition time or TR (effectively the fMRI ‘framerate’, which differs between 7 and 3 T scan sessions in the HCP) and averaged over all available fMRI sessions to produce a single scalar estimate of head motion in mm/min for each participant.

### 2.5 Statistical Methods

An important feature of the HCP dataset is the inclusion of twins and siblings. Of the 1037 participants who retained, these belong to only 431 families due to the inclusion of siblings. This includes 124 monozygotic twin pairs confirmed with genotyping data and a further 12 pairs identified by self‐report. Preliminary analysis revealed significant intra‐class correlation in both delay discounting and movement based on family structure, reinforcing the need for statistical inference methods suited to the sample.

We analysed both whole cortex average cortical thickness and whole cortex grey matter volume with linear mixed‐effects regression to account for family structure. Furthermore, we used the FSL PALM (Permutation Analysis of Linear Models) software to analyse cortical thickness on the entire cortical surface at a more granular level (per vertex of the FreeSurfer surface mesh) and within parcellated regions of the Desikan‐Killiany atlas. For all analyses we used the same set of covariates of no interest, described in detail below.

#### 2.5.1 Whole Cortex Analysis: Linear Mixed‐Effects Regression

For analysis of whole cortex average cortical thickness, we used a linear mixed‐effects regression. This allows coefficient estimates for the fixed effects such as head motion and age, which can be usefully compared to the results of prior studies.

Mixed‐effects regression is especially useful here because ordinary least squares regression assumes that samples are independent and identically distributed, an assumption violated within the HCP YA S1200 dataset. Linear mixed‐effects regression is designed for clustered data that accounts for the dependency of data by modelling a mixture of fixed and random effects—with fixed effects resembling conventional linear regression coefficients, and random effects accounting for variance due to clustered observations (Yu et al. 2022). In this case, a random intercept in the dependent variable (either cortical thickness or volume) was computed for each family. The modelling was performed using the *lme4* package for R, which uses restricted maximum likelihood estimation to compute the regression coefficients. The *anova* function included with *lme4* was used to assess the likelihood ratio of the full model with all covariates to reduced models with one of the covariates removed (the likelihood ratio test statistic). This ratio was then assessed against a Chi‐squared distribution with 1 degree of freedom for significance testing.

#### 2.5.2 Vertex‐Wise and Parcellated Region Analysis of Cortical Surface: PALM

Permutation Analysis of Linear Models (PALM) is a non‐parametric data permutation method that supports surface‐based data, including cortical thickness and is part of the FMRIB Software Library (FSL) (Jenkinson et al. 2012; Winkler et al. 2014). PALM does not require normality or independence of the input data and produces a null distribution for the test statistic by randomly shuffling the rows (observations) of the independent variables (either motion or delay discounting, and the other covariates listed in next section)—in this case producing 10,000 permutations of the dataset. These permutation datasets are then each used to fit a linear model for thickness at every vertex or parcellated region of the reconstructed cortical surface. The results of these models, which should be consistent with the null hypothesis due to the randomisation, are then used to determine the likelihood of the actually observed relationship between the independent variables and the thickness under the null hypothesis. The permutation test also permits exact control of the false positive rate, and the vertex‐wise results were family‐wise rate of error (FWER) corrected. Before fitting the model, the cortical thickness data was smoothed using a 15 mm full‐width half‐max (FWHM) surface kernel.

The presence of family structure (twins and other siblings) in the HCP S1200 violates the exchangeability assumption under which participants' data is usually shuffled. Specifically, when the sample is drawn from statistically dependent sub‐populations such as families, nested or multi‐level exchangeability blocks should be specified (Winkler et al. 2015) wherein shuffling only occurs between comparable groups (families of the same size/structure, and within families shuffling only between twins or non‐twin siblings).

The analysis was run using PALM alpha119 using MATLAB R2022a with exchangeability blocks generated from the script by Winkler et al. (2015); code available at https://fsl.fmrib.ox.ac.uk/fsl/docs/, allowing exchangeability between dizygotic twins and ordinary siblings. As PALM does not handle missing values, 29 participants were excluded due to missing datapoints, and 2 participants were excluded because their family relationship was not recorded, leaving 1006 participants. Variance groups were automatically assigned (i.e., homoscedasticity was assumed only within blocks) and variance groups of size 1 were removed (using ‘–removevgbysize 1’ option). Hence a further 176 participants were excluded from this analysis, bringing the sample down to 830.

To identify and plot regions of the cortical surface where significant effects were found (as in Figure 1C), threshold‐free cluster enhancement was used (Smith and Nichols 2009). However, to quantitatively evaluate the correlation between movement and delay discounting effects, we also compared the unthresholded statistical maps to each other using the spin‐permutation method devised by Alexander‐Bloch et al. (2018), a non‐parametric method for comparing surface maps which generates a null distribution by applying rotations to the surface map, hence quantifying the significance of correlation while accounting for the spatial extent and smoothness of the data.

In line with preliminary analysis that showed head motion primarily reducing estimated cortical thickness, we tested for a relationship between head motion and cortical thickness.

#### 2.5.3 Choice of Covariates

**FIGURE 2:**
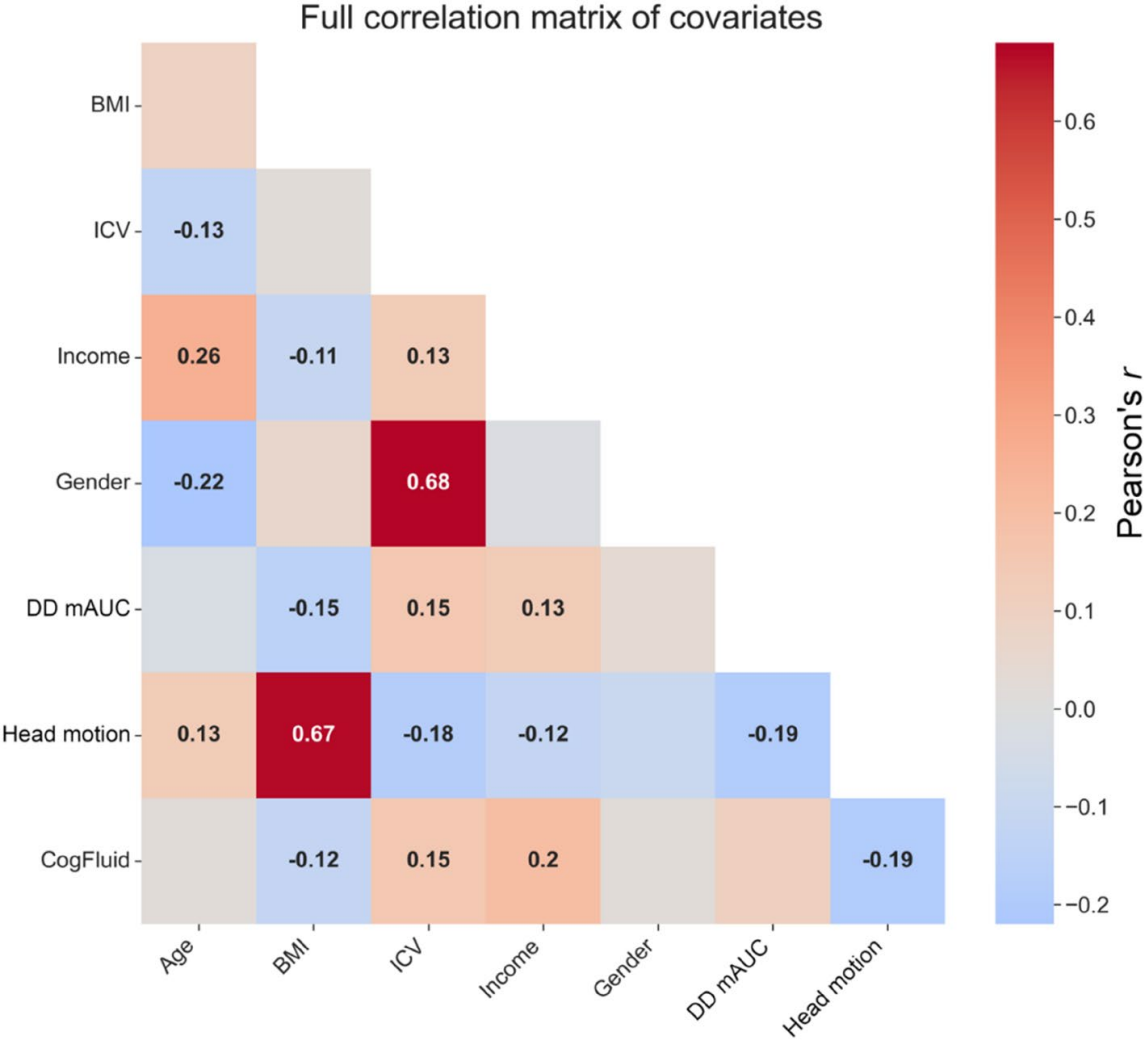
Correlation heatmap of the variables used in the regression models. Red colours indicate positive correlations, blue indicates negative correlations. Correlations with an absolute value greater than 0.1 are included as text annotations. Head motion = Average head motion derived from framewise displacement across all functional MRI scans measured in mm/min. Gender coded as Male = 1, Female = 0. BMI = body mass index; DD mAUC = delay discounting area under the curve averaged across $40,000 and $200 conditions; ICV = intracranial volume.

In the selection of covariates for the regression analysis (both mixed‐effects regression and PALM), variables were chosen on the basis of reported associations with brain structure and therefore possible confounding effects on cortical thickness. Age was included as numerous studies have documented its correlation with changes in brain structure, specifically reductions in cortical thickness and overall brain volume with age (Fjell et al. 2009; Kharabian Masouleh et al. 2022). The inclusion of gender as a covariate addresses reported sex differences in cortical thickness (Sowell et al. 2007). Intracranial volume (ICV) was included because it is the primary determinant of grey matter volume. Body Mass Index (BMI) is another important consideration, given its association with grey matter volume as reported by Taki et al. (2008), and its significant correlation with both in‐scanner movement and delay discounting behaviour (Figure 2). Age‐adjusted intelligence test scores, derived from the NIH Toolbox Cognition Battery fluid cognition composite, were included due to the reported link between IQ and cortical thickness (Narr et al. 2007; Schnack et al. 2015). Lastly, income (as measured by the 7‐point scale included in the Semi‐Structured Assessment for the Genetics of Alcoholism, SSAGA) was considered as a covariate due to its indirect association with brain structure and cognitive function, with results indicating altered trajectories of cortical thinning with age according to socioeconomic status (Piccolo et al. 2016).

## 3 Results

### 3.1 Correlation of Delay Discounting and In‐Scanner Movement

Head motion from the combined resting state and task‐based fMRI sessions correlates significantly with delay discounting mAUC (Pearson's *r* = −0.189, *p* = 1.49E‐09). When fitting a simple mixed‐effects regression to predict movement (i.e., allowing a random intercept for movement in each family and fitting a linear regression based only on delay discounting) the marginal *R* ^2^ (representing variance explained by the fixed effects; Nakagawa and Schielzeth 2013) was only 0.02, while the conditional R^2^ including the family effects was 0.31. This suggests that delay discounting does not explain a large amount of variance in movement beyond familywise clustering common to both head motion and delay discounting. By contrast, adding BMI to the model increased the marginal R^2^ to 0.45 and the conditional *R* ^2^ to 0.58, indicating that BMI explains a large amount of the variance in head motion observed both within and between families.

### 3.2 Whole‐Brain Average Cortical Thickness

The results of mixed‐effects regression on whole brain cortical thickness, including all covariates, delay discounting mAUC and fMRI‐estimated head movement are shown in Table 2. The estimated intercept of whole‐brain average cortical thickness is ~2.71 mm, and the most significant fixed effect by far is age, albeit only reducing thickness by 0.004 mm per year of life (over the age range of this sample, 22–37 year olds, replicating previous results cf. Vidal‐Pineiro et al. 2020). Secondly, BMI has a small positive impact of 0.002 mm per point of BMI. Delay discounting has no significant effect on whole‐brain cortical thickness, while head motion is associated with reduction in cortical thickness of 0.014 mm for every 1 mm/min of framewise displacement (over all resting‐state/task fMRI sessions).

**TABLE 2:** Results of mixed‐effects linear modelling for whole‐brain cortical thickness.

Fixed effect	Coef. estimate	Std. error	*t*‐value	Likelihood ratio (LR)	*p*(*χ* ^2^)
Intercept	2.71492	0.03821	71.047		
Age	**−0.0041**	**0.00075**	**−5.504**	**31.689**	1.8E−08***
Gender	−0.007	0.00715	−0.982	0.9834	0.3214
ICV	0.00427	0.00379	1.124	1.2944	0.2552
BMI	**0.00201**	**0.00064**	**3.139**	**9.9075**	0.0016**
Income	0.00254	0.00259	0.978	0.9665	0.3255
Fluid Cognition	0.00012	0.00022	0.552	0.2148	0.6431
DD mAUC	0.0033	0.00248	1.329	1.7784	0.1824
Head Motion	**−0.0137**	**0.00564**	**−2.434**	**6.0012**	0.0143*

*Note:* Effect sizes (coefficient estimates) are not normalised, that is, are presented in natural units for each variable unless otherwise stated. *p* values below 0.05 are highlighted in bold. * denotes significant *p* values below 0.05; ** denotes significant below 0.01; *** denotes significant below 0.001.

Abbreviations: Age: years; DD mAUC: average AUC from delay discounting task; Fluid Cognition: *Z*‐scored; Gender: 1 = male,0 = female; Head motion: mm/min; ICV: litres; Income: *Z*‐score of SSAGA 7‐point scale; Thickness: millimetres.

### 3.3 Whole‐Brain Cortical Grey Matter Volume

While most studies of brain morphology and delay discounting have examined cortical thickness, several studies have examined cortical grey matter volume also. As such, here we conducted comparable analyses using whole‐brain (total) volume (Table 3). Here we found that head motion had no impact on volume overall, while delay discounting mAUC did have a small but significant positive relationship—predicting greater cortical volume on average by ~7.8cm^3^ at the max mAUC value of 1 (i.e., indifference to delay).

**TABLE 3:** Results of mixed‐effects linear modelling for whole‐brain cortical grey matter volume.

Fixed effect	Coef. estimate	Std. error	*t*‐value	Likelihood ratio (LR)	*p*(*χ* ^2^)
Intercept	95.49	10.46	9.13		
Age	**−1.88**	**0.18**	**−10.49**	**104.26**	1.77E‐24***
Gender	**−4.01**	**1.73**	**−2.32**	**5.3009**	0.0213*
ICV	**0.33**	**6.23E‐3**	**53.41**	**1327.0**	1.53E‐290***
BMI	0.03	0.16	0.22	0.0478	0.8270
Income	0.11	0.63	0.17	0.0292	0.8643
Fluid Cognition	0.01	0.04	0.19	0.0350	0.8517
DD mAUC	**7.81**	**2.70**	**2.89**	**8.3284**	0.0039**
Head Motion	−0.25	1.37	−0.18	0.0329	0.8560

*Note:* Effect sizes (coefficient estimate) are not normalised, that is, are presented in natural units for each variable unless otherwise stated. *p* values below 0.05 are highlighted in bold. * denotes significant *p* values below 0.05; ** denotes significant below 0.01; *** denotes significant below 0.001.

Abbreviations: Age: years; DD mAUC: mean area under the curve from delay discounting task; Fluid Cognition: Z‐scored; Gender: 1 = male,0 = female; Grey matter volume: cubic centimetres; Head motion: mm/min; ICV: intracranial volume in cubic centimetres; Income: Z‐score of SSAGA 7‐point scale.

A comparable model was also conducted with cortical surface area, reported in Table S3.

### 3.4 Regional Cortical Thickness

Table 4 shows the results of running PALM for average cortical thickness over all regions of the Desikan‐Killiany parcellation, as visualised in Figure 1B. The regions listed are those significantly affected by motion before correction for multiple comparisons, but we also provide *p*‐values adjusted for false discovery rate (FDR, Benjamini and Hochberg 1995). The number of significant regions were found in the right hemisphere than the left (17 vs. 12). Three regions were significantly affected by delay discounting mAUC uncorrected, but none survived FDR correction, as shown in Table 5. The regions associated with delay discounting, except for right posterior cingulate cortex, were also significantly affected by head motion in our analysis. The results for all regions and both independent variables are contained in Table S1.

**TABLE 4:** Regions where head motion was significantly associated with reduced cortical thickness in parcellated PALM analysis.

	Region	*p*	*p*_FDR_
R	Temporal pole	**< 0.001**	**< 0.001**
L	Temporal pole	**< 0.001**	**0.007**
R	Entorhinal	**0.001**	**0.014**
L	Insula	**0.002**	**0.026**
R	Fusiform	**0.002**	**0.027**
R	Paracentral	**0.002**	**0.027**
L	Inferior temporal	**0.004**	**0.031**
L	Entorhinal	**0.004**	**0.031**
R	Precentral	**0.004**	**0.032**
L	Lateral orbitofrontal	**0.005**	**0.032**
R	Inferior temporal	**0.007**	**0.041**
R	Superior temporal	**0.008**	**0.044**
R	Transverse temporal	**0.009**	**0.046**
R	Cuneus	**0.012**	0.057
L	Precentral	**0.013**	0.057
R	Superior parietal	**0.014**	0.057
R	Inferior parietal	**0.015**	0.057
L	Transverse temporal	**0.015**	0.057
R	Precuneus	**0.019**	0.067
R	Insula	**0.023**	0.076
L	Superior temporal	**0.024**	0.076
R	Middle temporal	**0.025**	0.078
L	Pars opercularis	**0.036**	0.103
R	Supramarginal	**0.036**	0.103
R	Pars opercularis	**0.045**	0.115
L	Pars orbitalis	**0.046**	0.115
R	Lateral occipital	**0.046**	0.115
L	Lingual	**0.047**	0.115
L	Pars triangularis	**0.049**	0.115

*Note:* Regions shown were significant at the 5% level, either before or after FDR correction (Benjamini and Hochberg 1995). *p* values below 0.05 are highlighted in bold. Full results are given in Table S1.

**TABLE 5:** Regions where delay discounting mAUC was significantly associated with increased cortical thickness in parcellated PALM analysis.

	Region	*p*	*p*_FDR_
L	Entorhinal	**0.004**	0.177
R	Posterior cingulate	**0.005**	0.177
R	Fusiform	**0.023**	0.420

*Note:* Regions shown were only significant at the 5% level before FDR correction. Notably, all regions except the right precentral cortex were also found to be significantly affected by delay discounting, see Table 4. *p* values below 0.05 are highlighted in bold. Full results are presented in Table S1.

### 3.5 Regional Volume

We also performed PALM analysis on the volume of parcellated regions in the Desikan‐Killiany atlas, although no regions were found to be significantly related to delay discounting or to head motion after FDR correction. Regions where grey matter volume was significantly associated with head motion or delay discounting mAUC before correction for multiple corrections are included in Tables 6 and 7, respectively. The full results of the region volume analysis are contained in Table S2.

**TABLE 6:** Regions where head motion was significantly associated with reduced grey‐matter volume in parcellated PALM analysis.

	Region	*p*	*p*_FDR_
R	Paracentral	**0.001**	0.061
R	Temporal pole	**0.016**	0.502
R	Posterior cingulate	**0.023**	0.502
L	Temporal pole	**0.030**	0.502

*Note:* Regions shown were only significant at the 5% level before FDR correction. *p* values below 0.05 are highlighted in bold.

**TABLE 7:** Regions where delay discounting mAUC was significantly associated with grey‐matter volume in parcellated PALM analysis.

	Region	*p*	*p*_FDR_
L	Precentral	**0.012**	0.219
L	Middle temporal	**0.012**	0.219
L	Transverse temporal	**0.013**	0.219
L	Entorhinal	**0.013**	0.219
R	Entorhinal	**0.021**	0.269
R	Middle temporal	**0.024**	0.269
L	Postcentral	**0.030**	0.269
L	Temporal pole	**0.032**	0.269
R	Inferior temporal	**0.047**	0.313

*Note:* Regions shown were only significant at the 5% level before FDR correction. *p* values below 0.05 are highlighted in bold.

### 3.6 Vertex‐Wise Cortical Thickness

For greater sensitivity and granularity, we additionally analysed relationships between cortical thickness and head motion and delay discounting using a vertex‐wise analysis. Using FSL PALM, with exchangeability blocks respecting the family structure of the HCP S1200 (generated using the MATLAB code associated with Winkler et al. 2015), we checked for a positive relationship between delay discounting mAUC and cortical thickness (in other words, greater discounting associated with thinner cortex); the results are shown in Figure 1C. No clusters were found to be significantly affected by delay discounting (all *p* > 0.138). We also checked for a negative relationship between cortical thickness and head motion, also shown in Figure 1C. Large areas of cortex appear significantly thinner as the result of head movement, encompassing most of the regions where subthreshold effects were detected for delay discounting.

**FIGURE 3:**
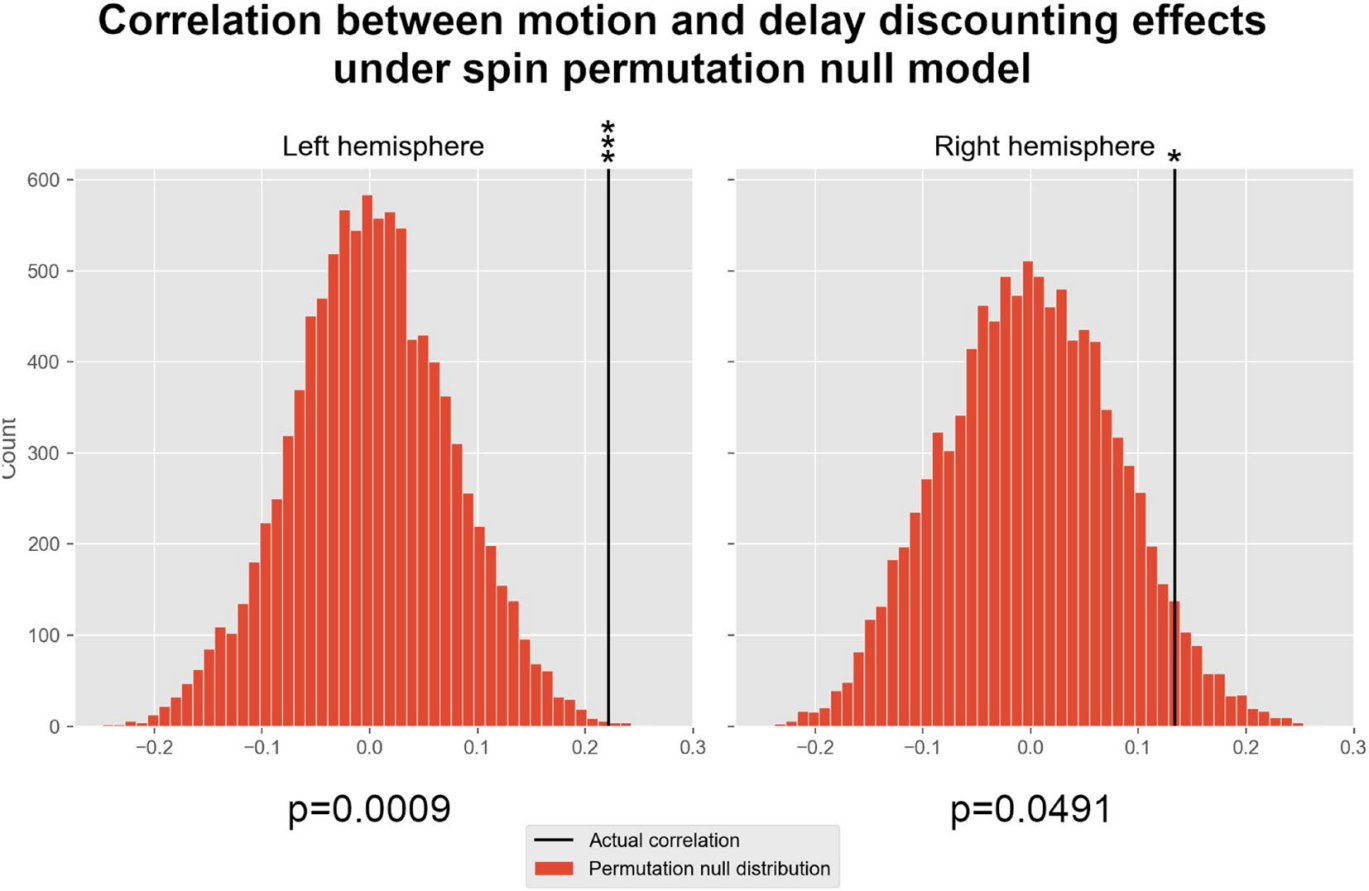
Correlation of effects under spin permutation null model. The histogram shows the frequency distribution of correlations for different randomised spins of the unthresholded, uncorrected statistical maps for delay discounting (mAUC) and head motion. The black line indicates the actually observed correlation when keeping the statistical maps in their original position. The probability values indicate the proportion of spin permutations that produced a higher correlation than was actually observed.

To produce a null distribution for assessing the significance of the correlation between the unthresholded statistical maps (data‐per‐vertex, ‘dpv’) we used spin permutations, results are shown in Figure 3. Almost no rotations (*p* = 0.0009) of the left hemisphere produced a stronger alignment of the effects than was actually observed, while the right hemisphere seems to correspond less closely, but was still significant (*p* = 0.0491) at the 5% significance level.

## 4 Discussion

### 4.1 Key Findings and Implications

Our analysis of the large HCP dataset found no significant effect of delay discounting on cortical thickness, either at the whole‐brain average level (Table 2), within parcellated cortical regions after FDR correction (Tables 5 and S2), or examining vertex‐wise data of the cortical surface (Figure 1C). This difference from previously reported findings detailed in Table 1 may be partly due to the larger sample size than previous studies. Although some of the studies reviewed used the same dataset (Garzón et al. 2023; Owens et al. 2017; Sadeh et al. 2023), only Garzón et al. (2023) used a method that takes into account family structure in the HCP sample (via PALM; Winkler et al. 2014, 2015), also finding no significant relation of delay discounting with cortical thickness. Nevertheless, the grey matter linked‐ICA component Garzón et al. identified as correlated with delay discounting was most heavily loaded in areas that appear severely affected by head‐motion artifacts, such as parts of the temporal gyrus (especially temporal pole) and sensorimotor cortex (cf. Figure 1B,C). We furthermore verified via spin permutation testing that the vertex‐wise reductions in cortical thickness associated with delay discounting in our analysis are significantly aligned with the (much stronger) head motion effects that we identified using the same method (Figure 3).

In contrast to the thickness results, whole‐brain analysis of cortical grey matter volume with the mixed‐effects regression found that delay discounting had a small but significant effect (positive with respect to AUC, meaning more discounting is associated with less volume), while head motion was not associated with altered volume despite reducing estimated cortical thickness, possibly due to increasing apparent surface area (Table S3). This delay discounting volume difference was not significant after FDR correction in any of the Desikan‐Killiany parcellated regions (see Table S2).

The results reported here illustrate the risk of motion bias in cortical morphometry estimates (Reuter et al. 2015). The movement effects discovered here are strong and more widespread than brain‐behaviour associations typically reported. They cover a broad range of specialised regions of cortex with little by way of common functional role, but which nonetheless have been implicated in many studies of delay discounting behaviour (cf. regions listed in Table 1). There is therefore a high risk that head motion has biassed the previous research findings linking delay discounting with cortical morphometry.

Our results stress the importance of accounting for motion‐related effects when identifying correlations between cortical morphometry and behavioural variables, such as delay discounting, that correlate with in‐scanner head movement, echoing earlier work by Kong et al. (2014) on movement confounds in fMRI studies of impulsivity. It is notable that cortical thinning effects of motion are easy to detect even in the presence of proper quality control measures and using only point estimates of head movement as the independent variable. In the worst‐case scenario, apparent differences in brain morphology associated with delay discounting and impulsivity may be entirely the result of differences in head movement (see also Madan 2018). Therefore, it is crucial to take measures to improve the reliability of brain‐behaviour associations discovered.

### 4.2 Limitations

The alignment between motion and delay discounting effects was barely significant in the right hemisphere (Figure 3), which may be connected to the hemispheric asymmetry observed in the motion and delay discounting effects (Figure 1B,C) which may result from some geometric distortion introduced by the registration to the symmetric FS_LR_32k mesh used in the HCP, or bias in the directionality of head motion (Figure S2).

Furthermore, a key limitation of the cross‐sectional observational dataset used here is that, although the results indicate significant similarity between the motion and delay discounting effects, we are unable to infer causality (i.e., that the delay discounting effects are in fact a side‐effect of its correlation with motion). Further work could in principle use simulations of head motion artifacts to demonstrate this (cf. Camara‐Rey et al. 2006; Olsson et al. 2024).

### 4.3 Suggestions for Further Research

Motion artifacts are always a potential problem for in vivo MRI studies. Extensive research has explored the problems caused by head motion in functional and diffusion‐weighted MRI (Oldham et al. 2020; Power et al. 2012; Yan et al. 2013). However, the same considerations apply for morphometry, especially of the cortex. In general, the standard ~1mm^3^ isotropic resolution acquired in most structural scans is not ideal for studying sub‐millimetre differences in the thickness of cortex that is highly convoluted and averages under 3 mm thick (see effect sizes in Table 2). Surface reconstruction methods such as FreeSurfer and CIVET perform remarkably well despite relatively low‐resolution input images, but it is becoming apparent that large sample sizes are needed to extract reliable structural brain‐behaviour correlations with current imaging technology (Kharabian Masouleh et al. 2019, 2022; Marek et al. 2022). However, high powered studies do not in themselves diminish the risk of spurious correlations as discussed here. Where possible, measures of head movement (e.g., from fMRI sessions [Alexander‐Bloch et al. 2016; Savalia et al. 2017] or perhaps from navigator volumes taken during the structural scan) should be checked for correlation with behavioural measures under study and should be included as nuisance variables in statistical analysis.

When taking this approach, it is necessary to include other covariates as well—head motion positively correlates significantly with both age and BMI, making it challenging to disentangle the true effect sizes attributable to each of the variables. Our results demonstrate that age and movement both predict reduced whole‐brain cortical thickness, suggesting that some of the apparent effect of age may be the result of head motion or vice versa. On the other hand, we showed that BMI appears to have a positive effect on estimated cortical thickness when controlling for movement, and therefore may reduce the apparent thinning effect due to head motion. This effect of BMI on head motion and cortical thickness may result from respiratory chest motion producing susceptibility variations in the B0 field (Madan 2018; Raj et al. 2001; Van de Moortele et al. 2002; van Gelderen et al. 2007).

A broader theoretical issue is that without reference to for example, neurobiological theories of how cortical thickness or volume influences functional dynamics (Breakspear 2017; Singer 2013), studies of cortical thickness relationships with delay discounting and other behavioural variables may be insufficiently hypothesis driven, and hence more susceptible to spurious findings (Oberauer and Lewandowsky 2019). Cortical thickness effect sizes associated with behaviour have been shown to be small (in relation to the overall thickness of the cortex, see effect sizes in Table 2 given in millimetres)—making it challenging to ascribe neurobiological significance. The observed small differences detectable in large samples may result from diverse underlying cytoarchitectonic and anatomical causes. To this end, it may be more beneficial to examine connections between cortical morphology and functional dynamics (especially with age; see Stier et al. 2023) as groundwork for understanding the behavioural implications of differences in brain morphology in more detail. Crucially, theories of cortical function typically make reference to laminar organisation rather than aggregate cortical thickness as a determining factor of function (Bastos et al. 2012; Douglas and Martin 2004), which cannot be analysed with the methods used here. However, as MRI spatial resolution and signal‐to‐noise ratio continue to improve, imaging the laminar organisation of the cortex should permit more specific conjectures about functional and behavioural consequences of structural variation to be tested (Saberi et al. 2023; Trampel et al. 2019).

Importantly when seeking to acquire detailed structural data, MRI sequences can be adapted to minimise the detrimental effects of head motion on image quality, but these methods are not yet standard. An emerging method for dealing with motion‐related artifacts in structural scans is prospective motion correction (Maclaren et al. 2013; Slipsager et al. 2022; Zaitsev et al. 2006), which applies real‐time correction for movement during the acquisition of MR images. Prospective motion correction can be done in at least two different ways: either by real‐time tracking of the participant's movement using external sensors (Frost et al. 2019; Lüsebrink et al. 2017; Maclaren et al. 2013), or by exploiting intrinsic data from the imaging device itself, such as navigator volumes in MRI (Dosenbach et al. 2017; Tisdall et al. 2012; White et al. 2010). Both approaches aim to reacquire or reparameterise motion‐affected data dynamically during the scan to minimise motion artifacts in the final volume. Prospective motion correction techniques have been shown to reduce error in cortical morphometry estimates and should reduce the risk of motion confounds in brain behaviour associations (Gil et al. 2024). If using navigator volumes, this approach does not require any additional hardware and only slightly increases scan time, only re‐acquiring segments when severe motion is observed. Future work may provide further insight on the specific patterns of distortion caused by head motion (Nárai et al. 2022), and machine learning methods may allow post hoc correction of distorted volumes (Iglesias et al. 2017; Spieker et al. 2024). Other approaches to the problem include physically reducing participant head motion with padding or tactile/visual feedback; for a review see Zaitsev et al. (2015). Yet another alternative is to include a movie to watch during structural scans where feasible, as this is known to reduce head motion versus no stimulus (Madan 2018).

In conclusion, the confound between delay discounting and in‐scanner head motion increases the likelihood of false‐positive findings in cortical thickness analyses of delay discounting. We suggest that measures to reduce or remediate head motion artifacts should be implemented for structural MRI studies wherever possible, and especially when behavioural measures of interest correlate with in‐scanner head movement as in the case of delay discounting. Where this is not possible, estimates of head movement derived from fMRI may be used as nuisance regressors for structural MRI analysis.

## Author Contributions

J.R.‐T., C.R.M. and M.S. conceptualised the study. J.R.‐T. conducted the initial literature review and performed the data analysis. All authors contributed to the writing, reviewed and approved the final manuscript.

## Funding

This research was supported by a BBSRC (Biotechnology and Biological Sciences Research Council) Doctoral Training Programme studentship at the University of Nottingham (Grant Reference: BB/T0083690/1). Data were provided by the Human Connectome Project, WU‐Minn Consortium (Principal Investigators: David Van Essen and Kamil Ugurbil; 1U54MH091657) funded by the 16 NIH Institutes and Centers that support the NIH Blueprint for Neuroscience Research and by the McDonnell Center for Systems Neuroscience at Washington University.

## Conflicts of Interest

The authors declare no conflicts of interest.

## Supporting information


**Figure S1:** Histograms of SPM vs. FreeSurfer derived intracranial volume (ICV) estimates, with height included for comparison. The FreeSurfer 5.2 ICV estimates have outliers on the left tail (volume underestimated) which can be seen as outliers on the 2D density plots with SPM estimates and height. Intracranial volume is measured in cubic millimetres, height is in inches.
**Figure S2:** Average movement over all sessions in the X, Y and Z directions is significantly different from zero (all *p* < 0.001). Rotational parameters were not significantly different from zero. This tendency to move asymmetrically may help explain the hemispheric asymmetry of cortical thinning motion artifacts (Figure 1B,C).
**Table S1:** Full results of region‐wise PALM analysis of cortical thickness. *p*‐values shown indicate the probability of the null hypothesis that cortical thickness measurements are not truly reduced by head motion or severity of delay discounting (‐mAUC). *p*‐values below 0.05 are highlighted with bold.
**Table S2:** Full results of region‐wise PALM analysis of cortical grey matter volume. *p*‐values shown indicate the probability of the null hypothesis that grey matter volume measurements are not truly reduced by head motion or severity of delay discounting (‐mAUC). *p*‐values below 0.05 are highlighted with bold.
**Table S3:** Results of mixed‐effects linear modelling for whole‐brain cortical surface area. Effect sizes (coefficient estimates) are not normalised, that is, are presented in natural units for each variable unless otherwise stated. Surface area: cm^2^; Age: years; Gender: 1 = Male,0 = Female; ICV: litres; Income: *Z*‐score of SSAGA 7‐point scale; Fluid Cognition: *Z*‐scored; DD mAUC: average AUC from delay discounting task; Head motion: mm/min.

## Data Availability

The data used in this study are publicly available as part of the Human Connectome Project Young Adults (S1200) release. To apply for access to this data please see the HCP website (https://humanconnectome.org). Code to replicate the results of this study is available as part of the *lme4* package (version 1.1–35.3) in R (version 4.4.1) and the PALM module (version alpha119) of the FMRIB Software Library. For the specific scripts written in this study, please contact the authors directly.
