# Supplementary material for: Bias in Structural MRI Correlates of Delay Discounting due to Head Motion
Source: Hum Brain Mapp. 2026 Feb 22;47(3):e70474. doi: 10.1002/hbm.70474 (PMC12928027; doi:10.1002/hbm.70474)
Supplement: Supplementary file 1 — Figure S1: Histograms of SPM vs. FreeSurfer derived intracranial volume (ICV) estimates, with height included for comparison. The FreeSurfer 5.2 ICV estimates have outliers on the left tail (volume underestimated) which can be seen as outliers on the 2D density plots with SPM estimates and height. Intracranial volume is measured in cubic millimetres, height is in inches. Figure S2: Average movement over all sessions in the X, Y and Z directions is significantly different from zero (all p < 0.001). Rotational parameters were not significantly different from zero. This tendency to move asymmetrically may help explain the hemispheric asymmetry of cortical thinning motion artifacts (Figure 1B,C). Table S1: Full results of region‐wise PALM analysis of cortical thickness. p‐values shown indicate the probability of the null hypothesis that cortical thickness measurements are not truly reduced by head motion or severity of delay discounting (‐mAUC). p‐values below 0.05 are highlighted with bold. Table S2: Full results of region‐wise PALM analysis of cortical grey matter volume. p‐values shown indicate the probability of the null hypothesis that grey matter volume measurements are not truly reduced by head motion or severity of delay discounting (‐mAUC). p‐values below 0.05 are highlighted with bold. Table S3: Results of mixed‐effects linear modelling for whole‐brain cortical surface area. Effect sizes (coefficient estimates) are not normalised, that is, are presented in natural units for each variable unless otherwise stated. Surface area: cm2; Age: years; Gender: 1 = Male,0 = Female; ICV: litres; Income: Z‐score of SSAGA 7‐point scale; Fluid Cognition: Z‐scored; DD mAUC: average AUC from delay discounting task; Head motion: mm/min. [file HBM-47-e70474-s001.docx]

# Supplementary Materials

**Figure S1:** Histograms of SPM vs FreeSurfer derived intracranial volume (ICV) estimates, with height included for comparison. The FreeSurfer 5.2 ICV estimates have outliers on the left tail (volume underestimated) which can be seen as outliers on the 2D density plots with SPM estimates and height. Intracranial volume is measured in cubic millimetres, height is in inches.


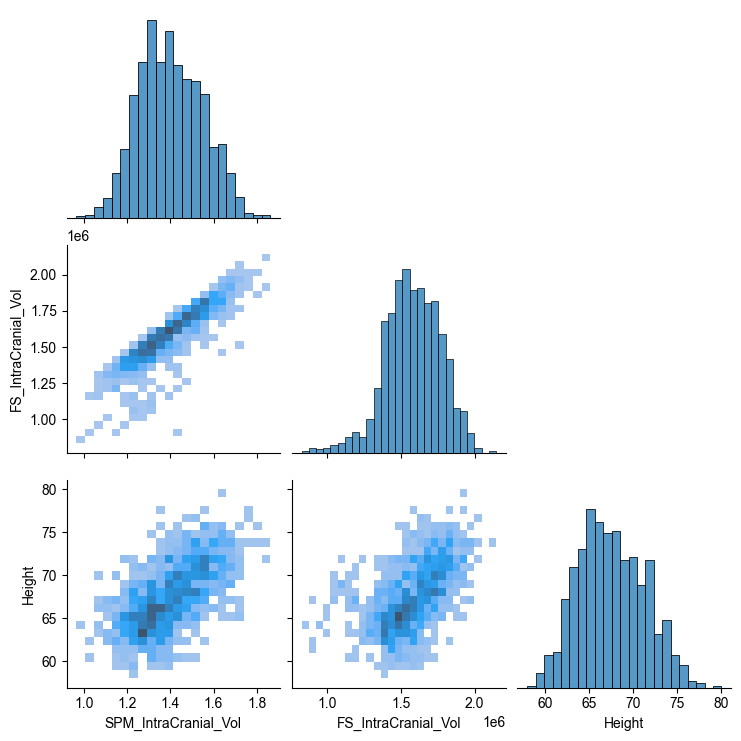


**Figure S2:** Average movement over all sessions in the X, Y, and Z directions is significantly different from zero (all p<.001). Rotational parameters were not significantly different from zero. This tendency to move asymmetrically may help explain the hemispheric asymmetry of cortical thinning motion artifacts (Figure 1B-C).


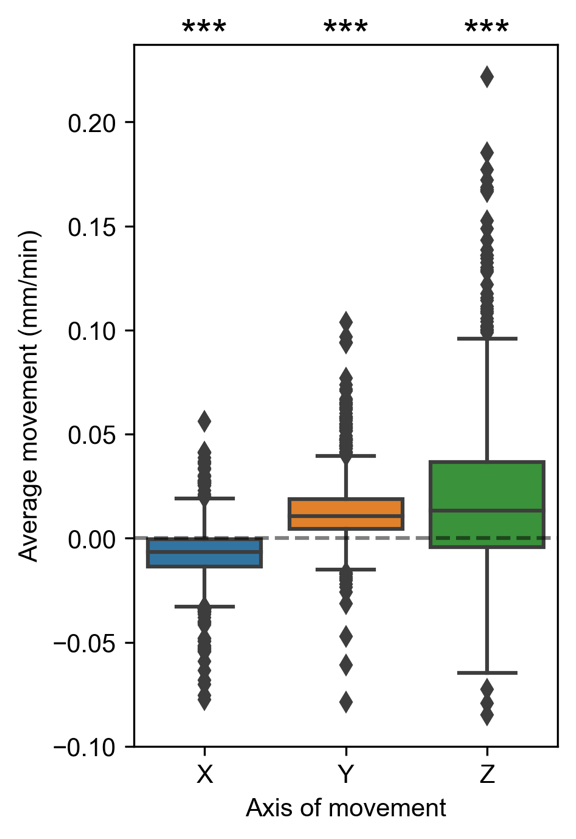


**Table S1:** Full results of region-wise PALM analysis of cortical thickness. p-values shown indicate the probability of the null hypothesis that cortical thickness measurements are not truly reduced by head motion or severity of delay discounting (-mAUC). p-values less than .05 are highlighted with bold text.

|  | | **Uncorrected p** | | **FDR Corrected p** | |
| --- | --- | --- | --- | --- | --- |
| **Hemi** | **Name** | **DDisc mAUC** | **Head Motion** | **DDisc mAUC** | **Head Motion** |
| L | Banks of superior temporal sulcus | 0.204 | 0.057 | 0.420 | 0.125 |
| L | Caudal anterior cingulate | 0.319 | 0.423 | 0.542 | 0.480 |
| L | Caudal middle frontal | 0.497 | 0.297 | 0.621 | 0.374 |
| L | Cuneus | 0.169 | 0.151 | 0.420 | 0.238 |
| L | Entorhinal | **0.004** | **0.004** | 0.177 | **0.031** |
| L | Fusiform | 0.081 | 0.080 | 0.420 | 0.145 |
| L | Inferior parietal | 0.475 | 0.311 | 0.621 | 0.384 |
| L | Inferior temporal | 0.148 | **0.004** | 0.420 | **0.031** |
| L | Isthmus cingulate | 0.435 | 0.182 | 0.605 | 0.257 |
| L | Lateral occipital | 0.098 | 0.347 | 0.420 | 0.412 |
| L | Lateral orbitofrontal | 0.083 | **0.005** | 0.420 | **0.032** |
| L | Lingual | 0.210 | **0.047** | 0.421 | 0.115 |
| L | Medial orbitofrontal | 0.532 | 0.851 | 0.623 | 0.864 |
| L | Middle temporal | 0.199 | 0.054 | 0.420 | 0.122 |
| L | Parahippocampal | 0.251 | 0.608 | 0.461 | 0.656 |
| L | Paracentral | 0.510 | 0.097 | 0.621 | 0.170 |
| L | Pars opercularis | 0.699 | **0.036** | 0.742 | 0.103 |
| L | Pars orbitalis | 0.075 | **0.046** | 0.420 | 0.115 |
| L | Pars triangularis | 0.198 | **0.049** | 0.420 | 0.115 |
| L | Pericalcarine | 0.797 | 0.163 | 0.821 | 0.244 |
| L | Postcentral | 0.426 | 0.077 | 0.605 | 0.145 |
| L | Posterior cingulate | 0.167 | 0.201 | 0.420 | 0.273 |
| L | Precentral | 0.356 | **0.013** | 0.584 | 0.057 |
| L | Precuneus | 0.235 | 0.138 | 0.457 | 0.228 |
| L | Rostral anterior cingulate | 0.739 | 0.551 | 0.774 | 0.605 |
| L | Rostral middle frontal | 0.204 | 0.394 | 0.420 | 0.454 |
| L | Superior frontal | 0.265 | 0.695 | 0.474 | 0.726 |
| L | Superior parietal | 0.699 | 0.297 | 0.742 | 0.374 |
| L | Superior temporal | 0.433 | **0.024** | 0.605 | 0.076 |
| L | Supramarginal | 0.173 | 0.194 | 0.420 | 0.269 |
| L | Frontal pole | 0.363 | 0.705 | 0.584 | 0.726 |
| L | Temporal pole | 0.445 | **0.000** | 0.605 | **0.007** |
| L | Transverse temporal | 0.142 | **0.015** | 0.420 | 0.057 |
| L | Insula | 0.127 | **0.002** | 0.420 | **0.026** |
| R | Banks of superior temporal sulcus | 0.148 | 0.067 | 0.420 | 0.143 |
| R | Caudal anterior cingulate | 0.177 | 0.352 | 0.420 | 0.412 |
| R | Caudal middle frontal | 0.523 | 0.148 | 0.623 | 0.238 |
| R | Cuneus | 0.511 | **0.012** | 0.621 | 0.057 |
| R | Entorhinal | 0.136 | **0.001** | 0.420 | **0.014** |
| R | Fusiform | **0.023** | **0.002** | 0.420 | **0.027** |
| R | Inferior parietal | 0.141 | **0.015** | 0.420 | 0.057 |
| R | Inferior temporal | 0.106 | **0.007** | 0.420 | **0.041** |
| R | Isthmus cingulate | 0.153 | 0.167 | 0.420 | 0.244 |
| R | Lateral occipital | 0.151 | **0.046** | 0.420 | 0.115 |
| R | Lateral orbitofrontal | 0.111 | 0.116 | 0.420 | 0.197 |
| R | Lingual | 0.289 | 0.081 | 0.504 | 0.145 |
| R | Medial orbitofrontal | 0.488 | 0.349 | 0.621 | 0.412 |
| R | Middle temporal | 0.097 | **0.025** | 0.420 | 0.078 |
| R | Parahippocampal | 0.185 | 0.221 | 0.420 | 0.294 |
| R | Paracentral | 0.159 | **0.002** | 0.420 | **0.027** |
| R | Parso percularis | 0.834 | **0.045** | 0.846 | 0.115 |
| R | Pars orbitalis | 0.679 | 0.168 | 0.742 | 0.244 |
| R | Pars triangularis | 0.611 | 0.273 | 0.691 | 0.356 |
| R | Pericalcarine | 0.980 | 0.077 | 0.980 | 0.145 |
| R | Postcentral | 0.419 | 0.080 | 0.605 | 0.145 |
| R | Posterior cingulate | **0.005** | 0.073 | 0.177 | 0.145 |
| R | Precentral | 0.620 | **0.004** | 0.691 | **0.032** |
| R | Precuneus | 0.378 | **0.019** | 0.584 | 0.067 |
| R | Rostral anterior cingulate | 0.443 | 0.866 | 0.605 | 0.866 |
| R | Rostral middle frontal | 0.242 | 0.641 | 0.457 | 0.681 |
| R | Superior frontal | 0.124 | 0.497 | 0.420 | 0.554 |
| R | Superior parietal | 0.554 | **0.014** | 0.638 | 0.057 |
| R | Superior temporal | 0.482 | **0.008** | 0.621 | **0.044** |
| R | Supramarginal | 0.130 | **0.036** | 0.420 | 0.103 |
| R | Frontal pole | 0.083 | 0.159 | 0.420 | 0.244 |
| R | Temporal pole | 0.375 | **0.000** | 0.584 | **0.000** |
| R | Transverse temporal | 0.121 | **0.009** | 0.420 | **0.046** |
| R | Insula | 0.060 | **0.023** | 0.420 | 0.076 |

**Table S2:** Full results of region-wise PALM analysis of cortical grey matter volume. p-values shown indicate the probability of the null hypothesis that grey matter volume measurements are not truly reduced by head motion or severity of delay discounting (-mAUC). p-values less than .05 are highlighted with bold text.

|  | | **Uncorrected p** | | **FDR Corrected p** | |
| --- | --- | --- | --- | --- | --- |
| **Hemi** | **Name** | **DDisc mAUC** | **Head Motion** | **DDisc mAUC** | **Head Motion** |
| L | Banks of superior temporal sulcus | 0.643 | 0.184 | 0.769 | 0.664 |
| L | Caudal anterior cingulate | 0.273 | 0.699 | 0.442 | 0.973 |
| L | Caudal middle frontal | 0.166 | 0.712 | 0.369 | 0.973 |
| L | Cuneus | 0.790 | 0.803 | 0.839 | 0.973 |
| L | Entorhinal | **0.013** | 0.126 | 0.219 | 0.614 |
| L | Fusiform | 0.194 | 0.171 | 0.397 | 0.664 |
| L | Inferior parietal | 0.645 | 0.465 | 0.769 | 0.928 |
| L | Inferior temporal | 0.155 | 0.054 | 0.369 | 0.548 |
| L | Isthmus cingulate | 0.088 | 0.917 | 0.313 | 0.973 |
| L | Lateral occipital | 0.052 | 0.904 | 0.313 | 0.973 |
| L | Lateral orbitofrontal | 0.081 | 0.125 | 0.313 | 0.614 |
| L | Lingual | 0.118 | 0.936 | 0.352 | 0.973 |
| L | Medial orbitofrontal | 0.579 | 0.355 | 0.743 | 0.862 |
| L | Middle temporal | **0.012** | 0.546 | 0.219 | 0.957 |
| L | Parahippocampal | 0.814 | 0.973 | 0.842 | 0.973 |
| L | Paracentral | 0.953 | 0.348 | 0.953 | 0.862 |
| L | Parso percularis | 0.327 | 0.736 | 0.494 | 0.973 |
| L | Pars orbitalis | 0.637 | 0.654 | 0.769 | 0.973 |
| L | Pars triangularis | 0.198 | 0.315 | 0.397 | 0.862 |
| L | Pericalcarine | 0.603 | 0.946 | 0.759 | 0.973 |
| L | Postcentral | **0.030** | 0.424 | 0.269 | 0.909 |
| L | Posterior cingulate | 0.678 | 0.073 | 0.795 | 0.548 |
| L | Precentral | **0.012** | 0.622 | 0.219 | 0.971 |
| L | Precuneus | 0.093 | 0.428 | 0.313 | 0.909 |
| L | Rostral anterior cingulate | 0.168 | 0.793 | 0.369 | 0.973 |
| L | Rostral middle frontal | 0.231 | 0.330 | 0.428 | 0.862 |
| L | Superior frontal | 0.176 | 0.477 | 0.373 | 0.928 |
| L | Superior parietal | 0.294 | 0.956 | 0.465 | 0.973 |
| L | Superior temporal | 0.162 | 0.276 | 0.369 | 0.862 |
| L | Supramarginal | 0.729 | 0.323 | 0.826 | 0.862 |
| L | Frontal pole | 0.233 | 0.541 | 0.428 | 0.957 |
| L | Temporal pole | **0.032** | **0.030** | 0.269 | 0.502 |
| L | Transverse temporal | **0.013** | 0.088 | 0.219 | 0.560 |
| L | Insula | 0.057 | 0.582 | 0.313 | 0.969 |
| R | Banks of superior temporal sulcus | 0.053 | 0.340 | 0.313 | 0.862 |
| R | Caudal anterior cingulate | 0.155 | 0.141 | 0.369 | 0.639 |
| R | Caudal middle frontal | 0.252 | 0.916 | 0.434 | 0.973 |
| R | Cuneus | 0.413 | 0.407 | 0.597 | 0.909 |
| R | Entorhinal | **0.021** | 0.163 | 0.269 | 0.664 |
| R | Fusiform | 0.097 | 0.666 | 0.313 | 0.973 |
| R | Inferior parietal | 0.156 | 0.058 | 0.369 | 0.548 |
| R | Inferior temporal | **0.047** | 0.091 | 0.313 | 0.560 |
| R | Isthmus cingulate | 0.875 | 0.838 | 0.888 | 0.973 |
| R | Lateral occipital | 0.312 | 0.819 | 0.482 | 0.973 |
| R | Lateral orbitofrontal | 0.069 | 0.070 | 0.313 | 0.548 |
| R | Lingual | 0.127 | 0.860 | 0.359 | 0.973 |
| R | Medial orbitofrontal | 0.817 | 0.549 | 0.842 | 0.957 |
| R | Middle temporal | **0.024** | 0.215 | 0.269 | 0.731 |
| R | Parahippocampal | 0.069 | 0.964 | 0.313 | 0.973 |
| R | Paracentral | 0.268 | **0.001** | 0.442 | 0.061 |
| R | Parso percularis | 0.253 | 0.416 | 0.434 | 0.909 |
| R | Pars orbitalis | 0.514 | 0.629 | 0.685 | 0.971 |
| R | Pars triangularis | 0.455 | 0.055 | 0.631 | 0.548 |
| R | Pericalcarine | 0.777 | 0.885 | 0.839 | 0.973 |
| R | Postcentral | 0.168 | 0.473 | 0.369 | 0.928 |
| R | Posterior cingulate | 0.527 | **0.023** | 0.689 | 0.502 |
| R | Precentral | 0.093 | 0.512 | 0.313 | 0.957 |
| R | Precuneus | 0.496 | 0.868 | 0.674 | 0.973 |
| R | Rostral anterior cingulate | 0.067 | 0.347 | 0.313 | 0.862 |
| R | Rostral middle frontal | 0.255 | 0.761 | 0.434 | 0.973 |
| R | Superior frontal | 0.074 | 0.860 | 0.313 | 0.973 |
| R | Superior parietal | 0.222 | 0.584 | 0.428 | 0.969 |
| R | Superior temporal | 0.790 | 0.611 | 0.839 | 0.971 |
| R | Supramarginal | 0.704 | 0.105 | 0.811 | 0.595 |
| R | Frontal pole | 0.119 | 0.185 | 0.352 | 0.664 |
| R | Temporal pole | 0.743 | **0.016** | 0.829 | 0.502 |
| R | Transverse temporal | 0.348 | 0.716 | 0.514 | 0.973 |
| R | Insula | 0.424 | 0.720 | 0.601 | 0.973 |

**Table S3:** Results of mixed-effects linear modelling for whole-brain cortical surface area. Effect sizes (coefficient estimates) are not normalized, i.e., are presented in natural units for each variable unless otherwise stated. Surface area: cm^2^; Age: years; Gender: 1=Male,0=Female; ICV: litres; Income: Z-score of SSAGA 7-point scale; Fluid Cognition: Z-scored; DD mAUC: average AUC from delay discounting task; Head motion: mm/min.

| **Fixed effect** | **Coef. Estimate** | **Std. Error** | **t-value** | **Likelihood Ratio (LR)** | **p(χ^2^)** |
| --- | --- | --- | --- | --- | --- |
| (Intercept) | 268.12 | 34.28 | 7.82 |  |  |
| Age | -2.92 | 0.58 | -5.01 | 24.74 | 6.55E-07 |
| Gender | -7.19 | 5.66 | -1.27 | 1.61 | 0.205 |
| ICV | 1,131.59 | 20.49 | 55.23 | 1,394.49 | 3.31E-305 |
| BMI | -1.75 | 0.50 | -3.48 | 12.03 | 5.23E-04 |
| Income | -1.94 | 2.03 | -0.95 | 0.91 | 0.341 |
| Fluid Cognition | -0.08 | 0.12 | -0.66 | 0.44 | 0.509 |
| DD mAUC | 11.76 | 8.70 | 1.35 | 1.82 | 0.177 |
| Head Motion | 9.18 | 4.42 | 2.08 | 4.30 | 0.038 |

## Appendix: Establishing reliability of the framewise displacement metric

To establish the validity of using FD measurements from the various functional MRI sessions as a proxy for structural MRI session head motion, we computed the intra-class correlations (ICC) across sessions. ICC is a statistical measure commonly used to assess the reliability or agreement of quantitative measurements made by different observers, instruments, or under different conditions (Shrout and Fleiss, 1979). ICC measures the proportion of total variance in the measurements that is attributable to variance between groups as opposed to variance within groups.

In this case, we wish to quantify the variation in FD between participants as opposed to within participants (as judged across multiple fMRI sessions). A high ICC suggests that most of the variability is due to true differences between participants, not noise or inconsistencies between sessions (e.g., task vs resting state). There are different types of ICC depending on the model and interpretation needed. Here, we report two key values: ICC(1,k) and ICC(1,1).

The formula for ICC(1,k) is: $ICC(1,k) =\frac{BMS - WMS}{BMS}$

Where *BMS* is the mean squared deviation between participants (i.e., the variance between participants’ average FD values). *WMS* is the mean squared deviation within participants (i.e., the variance between scan sessions of a single participant).

The formula for ICC(1,1) is: $ICC(1,1)=\frac{BMS - WMS}{BMS + (k - 1)WMS}$

Where *k* is the number of judges rating each target (Shrout and Fleiss, 1979), in this case therefore, the number of scans available for each participant.

We computed ICC using the *pingouin* Python library, which estimates ICC by fitting a two-way random effects ANOVA model (factors: participant and session).

ICC(1,1) measures the reliability of a single session's FD value for an individual and was relatively low (0.167). As such, any single session's FD measurement is a noisy estimate of that participant's expected head motion in another session. Some fluctuation across sessions is expected due to momentary or situational factors.

ICC(1,k) measures the reliability of the average FD across multiple sessions for an individual, and was much higher (0.864). This suggests that when averaging across sessions, the estimate of a participant’s typical head motion becomes much more reliable; over 86% of the variance in session-wise FD values was therefore explained by stable individual differences rather than session-to-session fluctuations.

The difference between the ICC(1,1) and ICC(1,k) indicates that averaging over multiple sessions provides a more appropriate estimate of an individual’s trait-like tendency to in-scanner motion, as may correlate with other behavioural and physical traits. Thus, in the absence of motion-tracking data from the structural scans themselves, we chose to use the average FD over all available fMRI sessions to estimate the magnitude of structural scan head motion.

**References**

Shrout, P. E., & Fleiss, J. L. (1979). Intraclass correlations: uses in assessing rater reliability. *Psychological Bulletin*, 86(2), 420-428. [https://doi.org/10.1037/0033-2909.86.2.420](https://psycnet.apa.org/doi/10.1037/0033-2909.86.2.420)
